# Depleted before the dose: the mitochondrial vulnerability threshold hypothesis for fluoroquinolone-associated multisystem toxicity

**DOI:** 10.3389/fphar.2026.1905942

**Published:** 2026-09-23

**Authors:** Patrick E. Sewell, Christopher J. Jensen

**Affiliations:** Triple Helix Science, Santa Ana, CA, United States

**Keywords:** acquired mitochondrial disease, fluoroquinolone, fluoroquinolone-associated multisystem toxicity, integrative and regenerative pharmacology, mitochondrial reserve capacity, mitochondrial toxicity, mitochondrial vulnerability threshold, tendinopathy

## Abstract

Fluoroquinolone (FQ) antibiotics have an established capacity for mitochondrial injury, yet only a subset of exposed patients develop severe or persistent toxicity. We propose the Mitochondrial Vulnerability Threshold (MVT) hypothesis: that whether a standard therapeutic FQ dose produces clinically significant toxicity may depend substantially on the patient’s pre-existing mitochondrial reserve capacity rather than on fixed pharmacological properties alone. Although this toxicity was first recognized through musculoskeletal injury—particularly tendinopathy and tendon rupture—the reported syndrome extends to peripheral nerve, autonomic, central nervous system, and other tissues; we therefore use the term fluoroquinolone-associated multisystem toxicity (FQMT), with musculoskeletal (tendon) injury as its prototypical and best-characterized manifestation. We hypothesize that converging iatrogenic, nutritional, infectious, lifestyle, and environmental stressors—statin-associated CoQ10 depletion, proton-pump-inhibitor magnesium and B12 malabsorption, metformin-associated B12 depletion with genetically stratifiable risk, physical inactivity, metabolic syndrome, post-COVID-19 mitochondrial injury, antibiotic-induced dysbiosis, and environmental toxin burden—may contribute to higher apparent vulnerability in contemporary prescribing populations. We do not claim to demonstrate a temporal increase in FQMT incidence or severity; this is presented as a testable proposition. We further outline, as a speculative and explicitly unvalidated mechanism, whether epigenetic modification of the mitochondrial DNA displacement loop (D-loop) could contribute to escalating toxicity on repeat exposure. We propose the Mitochondrial Vulnerability Score (MVS) as a candidate research instrument (not a clinical prescribing tool) and present five falsifiable predictions with study designs. We discuss, with explicit caution, whether severe FQMT might in future be considered an acquired mitochondrial disorder, and we specify the evidence that such a classification would require. All clinical, therapeutic, and prophylactic content is hypothesis-generating and requires prospective validation.

## Graphical Abstract

**Figure:**
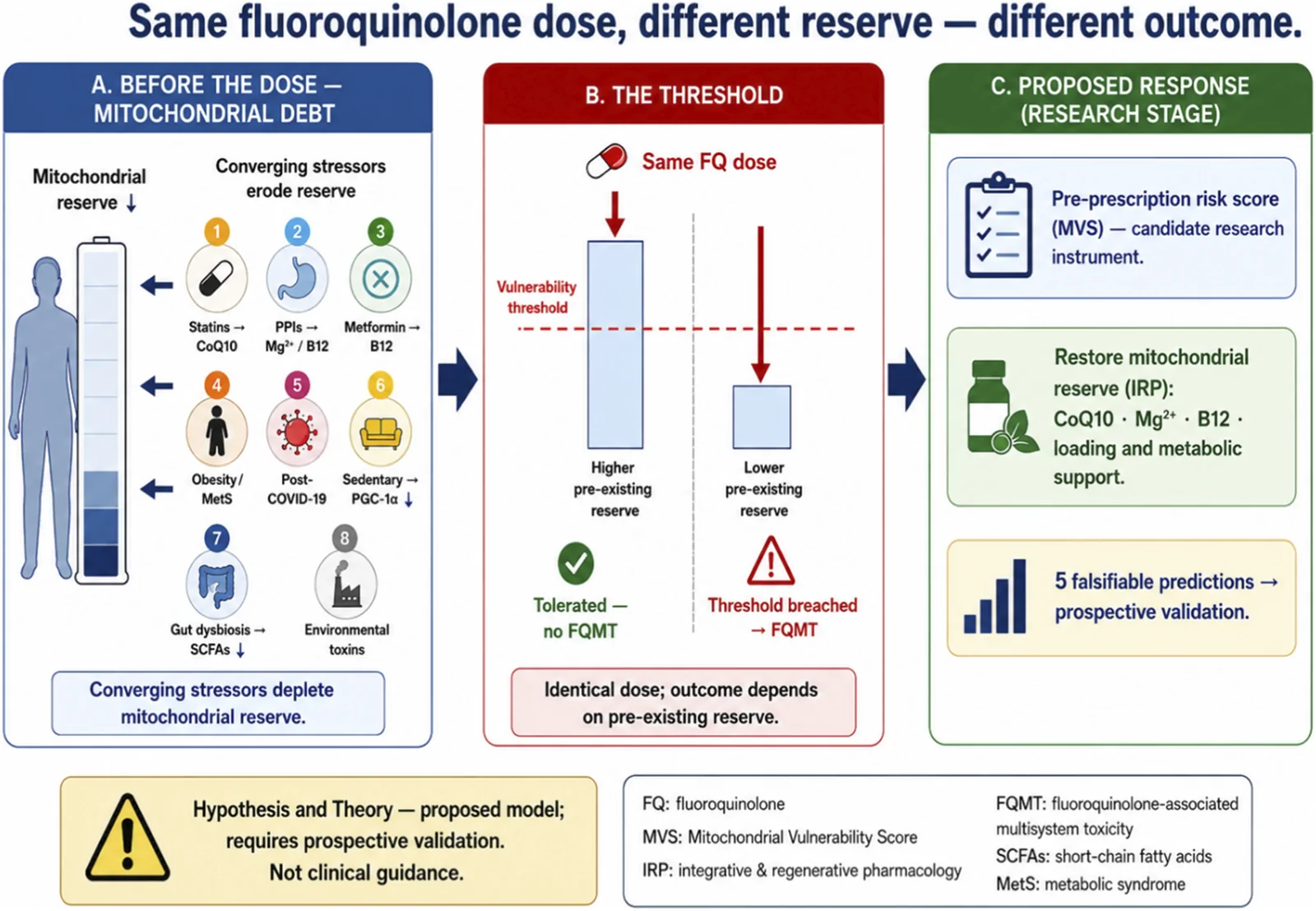
We propose that fluoroquinolone-associated multisystem toxicity (FQMT) emerges when a fixed fluoroquinolone insult exceeds a patient’s already-depleted mitochondrial reserve. Converging iatrogenic, nutritional, infectious, and environmental stressors are hypothesized to erode reserve, so that the same dose is more likely to breach the Mitochondrial Vulnerability Threshold. A candidate pre-prescription instrument (the Mitochondrial Vulnerability Score) and reserve-restoration strategies are proposed, with five falsifiable predictions for prospective validation. Conceptual/proposed model—not clinical guidance.

## Introduction

Fluoroquinolone (FQ) antibiotics—including ciprofloxacin, levofloxacin, and moxifloxacin—are among the most widely prescribed antimicrobial agents globally. Since 1995 this drug class has carried FDA regulatory warnings for tendinopathy and tendon rupture, expanded in 2008 and 2016 to encompass peripheral neuropathy and central nervous system effects (Bratsman et al., 2020). A nationwide Taiwanese cohort study (99% national health-insurance coverage; median follow-up 11 years) reported an adjusted hazard ratio of 1.42 (95% CI 1.02–1.87) for tendon disorders in FQ-exposed versus unexposed patients, with amplified risk in those co-prescribed statins, glucocorticoids, or aromatase inhibitors, and in those with chronic kidney disease, diabetes, or obesity (Chang et al., 2022).

A 2024 analysis of 61.7 million United States administrative claims documented a tendinopathy/tendon-rupture prevalence of 3.4% in patients with atherosclerotic cardiovascular disease (ASCVD) versus 1.9% in the general population—with 67.9% of ASCVD patients on statins and 16.7% on fluoroquinolones (Gillard et al., 2024). Despite these signals, the 2016 expanded FDA boxed warning was not followed by a statistically significant reduction in FQ prescription rates for the three indications it addressed (Bratsman et al., 2020); inappropriate prescribing for uncomplicated cystitis has been reported at 89.5% (Robinson et al., 2019); and a 2025 multimodal Veterans Affairs stewardship intervention achieved only partial reductions despite intensive pharmacist effort (Kinyua et al., 2025).

Pharmacovigilance analyses of the FDA Adverse Event Reporting System (FAERS, 2011–2024) have generated disproportionality signals for psychiatric and ocular events across multiple FQ agents (Chau and Au, 2026), an approximately six-fold higher reporting of panic attacks versus azithromycin (Raguram et al., 2026), and a broad reported toxicity profile spanning cardiotoxicity, aortic aneurysm, nephrotoxicity, neuropathy, and CNS effects (Hussen et al., 2024; Radkowski et al., 2025). These pharmacovigilance data warrant an important interpretive caution that we apply throughout this manuscript: FAERS disproportionality can generate hypotheses and flag potential safety signals, but—because it is a spontaneous reporting system without a defined exposure denominator and is subject to stimulated reporting, reporting bias, and secular changes in clinical awareness—it cannot establish incidence, risk magnitude, temporality, or causality, and an expansion of reported signals over time cannot be taken as evidence that the underlying biological severity of FQMT has increased.

Against this backdrop, a clinically important question remains incompletely addressed: why do some patients develop severe or persistent FQMT whereas many others tolerate the same drug class uneventfully? We propose that part of the answer lies not only in the drug but in the host—specifically, in inter-individual differences in mitochondrial reserve. This paper approaches the problem from the perspective of integrative and regenerative pharmacology (IRP): rather than asking only why the drug is harmful, we ask why a given patient may be more vulnerable, and what multi-level, restoration-oriented strategies might rebuild the biological reserve that ordinarily prevents harm.

This paper presents the Mitochondrial Vulnerability Threshold (MVT) hypothesis: that FQMT manifests when the drug’s acute mitochondrial insult exceeds an already-compromised bioenergetic reserve. We review the mechanistic evidence for each proposed contributing stressor, present five falsifiable predictions, introduce the Mitochondrial Vulnerability Score (MVS) as a candidate research instrument, and discuss—with explicit caution—whether severe FQMT might in future be considered an acquired mitochondrial disorder. Every clinical inference derived from this framework should be regarded as hypothesis-generating until prospectively validated.

## Scope, terminology, and literature-search strategy

### Scope and terminology

The FDA warnings and the earliest mechanistic work concern musculoskeletal injury, but the reported clinical picture is broader, and confining the hypothesis to tendon biology alone would understate the phenomenon it seeks to explain. We therefore adopt the term fluoroquinolone-associated multisystem toxicity (FQMT) and treat musculoskeletal (tendon) injury as the prototypical, best-characterized, and most mechanistically established manifestation, while explicitly distinguishing the strength of evidence across affected systems. Where a statement pertains specifically to tendon or muscle, we say so.

### Literature-search strategy

This is a Hypothesis and Theory article and constitutes a narrative synthesis rather than a systematic review. We searched PubMed/MEDLINE and Google Scholar (inception to March 2026) using combinations of the terms “fluoroquinolone”, “ciprofloxacin”, “levofloxacin”, “moxifloxacin”, “tendinopathy”, “tendon rupture”, “mitochondria”, “mitochondrial toxicity”, “oxidative stress”, “CoQ10”, “magnesium”, “vitamin B12”, “PGC-1α”, “D-loop methylation”, “long COVID”, and “acquired mitochondrial disease”, supplemented by hand-searching of reference lists and by FAERS-based pharmacovigilance reports. We intentionally emphasized (i) direct mechanistic studies of FQ mitotoxicity in connective-tissue cells, (ii) pharmacoepidemiological cohort and claims data on tendon and multisystem outcomes, and (iii) evidence bearing on each proposed host stressor. Because the framework is integrative, we drew on adjacent literature (statin, PPI, metformin, post-viral, and environmental mitochondrial toxicity) that are not FQ-specific; these are labeled as such. Contradictory or counterbalancing evidence—including mixed clinical trial data for CoQ10, the limitations of serum versus tissue magnesium measurement, the uncertain clinical relevance of mtDNA methylation, and non-mitochondrial mechanisms of FQ toxicity—is considered explicitly. We did not apply formal systematic-review inclusion criteria or risk-of-bias scoring, which is a limitation.

## The mitochondrial vulnerability threshold (MVT) hypothesis

We define the Mitochondrial Vulnerability Threshold as the level of pre-existing mitochondrial impairment above which a standard therapeutic FQ dose is hypothesized to produce clinically significant toxicity that would not occur in a patient with normal mitochondrial function. The concept draws on the established biology of mitochondrial threshold effects: cells can sustain substantial mitochondrial dysfunction before functional failure becomes clinically manifest, because oxidative-phosphorylation capacity normally exceeds resting physiological demand—the bioenergetic reserve, or spare respiratory capacity. Recent work demonstrates that mitochondrial DNA haplogroup variation shapes individual susceptibility to drug-induced mitotoxicity even against a constant nuclear background (Jensen et al., 2025), establishing that heterogeneity in mitochondrial reserve is a measurable pharmacogenomic parameter.

Throughout this manuscript we use two related terms. Mitochondrial reserve capacity refers to the spare bioenergetic and antioxidant capacity of a tissue—operationally, parameters such as spare respiratory capacity, maximal and coupled oxygen-consumption rate, mtDNA copy number, electron-transport-chain complex activities, tissue CoQ10 and glutathione content, and PGC-1α–driven biogenic potential. Mitochondrial reserve restoration refers to interventions intended to raise those same measurable parameters toward normal—for example, repleting depleted electron-transport substrates (CoQ10), restoring antioxidant buffering (glutathione via N-acetylcysteine), correcting cofactor deficiencies (magnesium, B12), and stimulating biogenesis (graded loading via PGC-1α). We do not use “restoration” to imply a validated clinical effect; we use it to name the mechanistic target and the functional parameters that a successful intervention would be expected to move.

The MVT hypothesis makes three claims, each stated as a testable proposition rather than an established finding: (1) mitochondrial reserve capacity varies substantially between individuals and may, on average, be lower in contemporary prescribing populations than in earlier decades, owing to convergent iatrogenic, nutritional, infectious, and environmental stressors; (2) lower reserve is hypothesized to make it more likely that a standard FQ dose breaches the threshold for clinical FQMT; and (3) prior FQ exposure may further lower the threshold, potentially via epigenetic modification of mitochondrial DNA. We emphasize that claim (1) is a hypothesis, not a demonstrated temporal trend: we have not performed, and do not cite, formal epidemiological trend analyses using standardized FQMT case definitions and prescription denominators, and none to our knowledge exist. The comparison between an earlier and a contemporary “average patient” used in Figure 1 is a conceptual device to illustrate the threshold model, not an empirically measured contrast.

**FIGURE 1:**
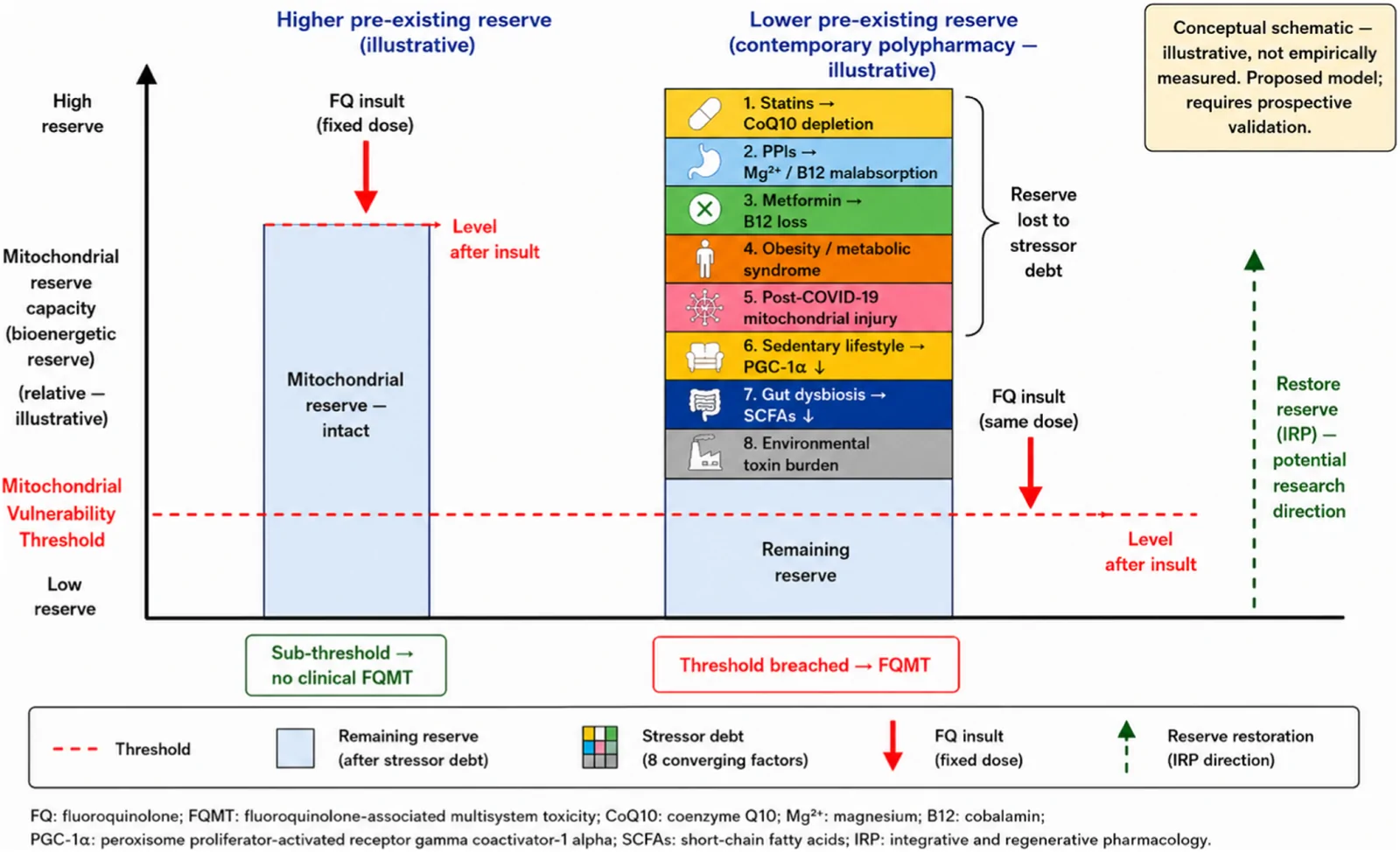
Conceptual schematic of the mitochondrial vulnerability threshold (illustrative—not empirically measured). The diagram illustrates the threshold model: where pre-existing mitochondrial reserve is high, an acute FQ insult is hypothesized to remain sub-threshold and produce no clinical FQMT; where reserve has been eroded by converging stressors, the same drug dose is hypothesized to breach the threshold and produce FQMT. The two patient states shown are illustrative devices, not measured reserve values, and the earlier-versus-contemporary contrast is conceptual rather than an empirically quantified comparison. An IRP framework suggests that supporting mitochondrial reserve is a potential research direction alongside prescribing reform. Proposed model—requires prospective validation. FQ: fluoroquinolone; FQMT: fluoroquinolone-associated multisystem toxicity; CoQ10: coenzyme Q10; PGC-1α: peroxisome proliferator-activated receptor gamma coactivator 1-alpha; SCFAs: short-chain fatty acids.

### Relationship to existing models of FQ toxicity

Prior mechanistic accounts of FQ toxicity are largely drug-centered: they characterize intrinsic properties of the molecule—topoisomerase-II/gyrase homology and mtDNA effects, chelation of divalent cations (notably Mg^2+^), reactive-oxygen-species generation, matrix-metalloproteinase activation, and tenocyte apoptosis (Lowes et al., 2009; Pouzaud et al., 2004; Bujor et al., 2013) — and treat host factors such as age, renal function, and corticosteroid co-exposure as independent risk modifiers. The MVT hypothesis does not replace these mechanisms; it situates them within a host-centered model. Its distinguishing claim is that the same fixed drug insult produces divergent clinical outcomes because it is superimposed on a variable, and potentially modifiable, host bioenergetic reserve. This host-centered, reserve-threshold model yields three features absent from purely drug-centered accounts: it integrates otherwise disparate risk factors (co-medications, comorbidity, infection, lifestyle, environment) into a single quantitative construct (cumulative mitochondrial debt); it predicts specific, testable dose–reserve interactions; and it points toward restoration of reserve—not only avoidance of the drug—as a therapeutic and preventive strategy.

### Which mechanisms apply to which affected system

Because we adopt a multisystem definition, we make explicit which mechanisms are best supported for each tissue, and at what level of evidence. This mapping is intended to prevent the strength of tendon-specific data from being read as if it applied equally to nerve or CNS.

Musculoskeletal (tendon, muscle) — best characterized. Direct *in vitro* evidence in human Achilles tenocytes demonstrates FQ-induced reactive-oxygen-species generation, loss of mitochondrial membrane potential, and apoptosis, with superior protection from the mitochondria-targeted antioxidant MitoQ (Lowes et al., 2009); Mg^2+^ chelation destabilizes collagen fibrils (Rosanoff et al., 2016); and matrix-metalloproteinase upregulation drives collagen degradation (Bujor et al., 2013). Pharmacoepidemiological data (Chang et al., 2022; Gillard et al., 2024; Koumou et al., 2021) support clinical tendon risk and its modification by comorbidity and co-medication.

Peripheral nerve and autonomic—moderate, largely clinical/pharmacovigilance. Peripheral neuropathy is FDA-recognized and reported in reviews of long-term FQ toxicity (Hussen et al., 2024; Radkowski et al., 2025); mechanistically it is attributed to a combination of mitochondrial oxidative injury in metabolically demanding neurons and, potentially, cofactor (B12, magnesium) insufficiency, but direct human mechanistic data are more limited than for tendon.

Central nervous system and psychiatric—mechanistically distinct in part; signal-level evidence. FQ CNS effects are attributed substantially to a non-mitochondrial pharmacodynamic mechanism—GABA-A receptor antagonism by the piperazinyl substituent with NMDA activation, potentiated by NSAIDs (Radkowski et al., 2025) — in addition to any mitochondrial contribution. Psychiatric and ocular signals derive primarily from FAERS disproportionality analyses (Chau and Au, 2026; Raguram et al., 2026) and therefore carry the interpretive limitations.

General mitochondrial mechanisms—cross-cutting. ROS generation, electron-transport-chain disruption, mtDNA effects, and depletion of shared substrates (CoQ10, glutathione, magnesium, B12) are common to all high-energy tissues and provide the unifying rationale for a reserve-threshold model, but their tissue-specific clinical consequences differ and are established to differing degrees, as above.

### The mitochondrial mechanism of FQMT: updated evidence base

Understanding the intrinsic mitochondrial toxicity of fluoroquinolones is prerequisite to examining how host vulnerability might amplify this harm. Table 1 summarizes established and emerging mechanisms, with the level of evidence indicated for each.

**TABLE 1:** Mechanisms of fluoroquinolone-associated musculoskeletal and neurological toxicity, with primary affected system and evidence level. Direct *in vitro* = demonstrated in human/mammalian cells; mechanistic/indirect = inferred from related systems or pharmacology; signal-level = pharmacovigilance disproportionality only.

Mechanism	Molecular pathway	Affected system/evidence level	Clinical consequence + reference
Topoisomerase II inhibition	Structural homology between bacterial DNA gyrase and mammalian mitochondrial Top2β; FQs inhibit both at therapeutic concentrations	Mitochondrial (general); mechanistic	mtDNA replication stress; tenocyte apoptosis (Golomb et al., 2015)
Mg^2+^ chelation	FQ keto/carboxyl groups form stable chelates with divalent cations; tissue Mg^2+^ bound independent of serum levels	Tendon/connective tissue; mechanistic + indirect clinical	Collagen fibril instability; tendon microrupture (Rosanoff et al., 2016)
Mitochondrial ROS generation	ETC disruption → superoxide; ∼3-fold ROS elevation in human Achilles tenocytes; MitoQ superior to non-targeted antioxidant in direct *in vitro* comparison	Tendon; direct *in vitro*	Tenocyte lipid peroxidation; membrane damage (Lowes et al., 2009)
MMP upregulation	Mg^2+^ loss + oxidative stress activate MMP-1, -3, -8, -13; Erk1/2 pathway involvement shown for ciprofloxacin	Tendon/connective tissue; direct *in vitro* (fibroblast)	Collagen I/III degradation; self-amplifying proteolysis (Bujor et al., 2013)
Tenocyte apoptosis	Cytochrome c release; caspase-3 activation via intrinsic mitochondrial pathway	Tendon; direct *in vitro*	Net tenocyte loss; impaired matrix repair (Lowes et al., 2009)
GABA-A antagonism + NMDA activation (non-mitochondrial)	FQ piperazinyl substituents antagonize GABA-A receptors; NMDA activation; NSAIDs potentiate pharmacodynamically	CNS/peripheral nerve; mechanistic + signal-level	CNS/neuropathy effects; panic-attack reporting ∼6-fold vs. comparators, FAERS (Radkowski et al., 2025; Raguram et al., 2026)

MMP: matrix metalloproteinase; ROS: reactive oxygen species; GABA: gamma-aminobutyric acid; FAERS: FDA adverse event reporting system; ROR: reporting odds ratio.

The study by Lowes et al. (2009) remains the most mechanistically definitive evidence for FQ-induced tenocyte mitochondrial damage: both ciprofloxacin and moxifloxacin produced up to a three-fold ROS increase and loss of mitochondrial membrane integrity in human Achilles tenocytes, with mitochondria-targeted MitoQ providing superior protection over a non-targeted antioxidant—directly implicating the mitochondrial compartment. Ciprofloxacin also modulates DNA methyltransferase 1 (Dnmt1) expression in fibroblasts through an Erk1/2-dependent mechanism (Bujor et al., 2013), suggesting FQ-induced epigenetic effects in connective-tissue cells that extend beyond the mitochondrion.

Consistent with a threshold model, a population-based cohort of 4.4 million adolescents found only small excess risks of tendon rupture (1.9 per 100,000) with FQ versus comparator antibiotics (Ross et al., 2021) — a group with high mitochondrial reserve and few of the co-medication burdens. By contrast, the Taiwan cohort demonstrated markedly amplified hazards in patients co-prescribed statins or corticosteroids or with chronic kidney disease (Chang et al., 2022), and the ASCVD claims data showed high co-medication rates in the highest-prevalence group (Gillard et al., 2024). We note these are observational associations consistent with—but not proof of—a mitochondrial threshold mechanism.

## The mitochondrial debt: eight converging population-level stressors

We identify eight categories of pre-existing mitochondrial impairment that may constitute a “mitochondrial debt” in some patients. Each is supported by independent literature; their convergence in the same individual—increasingly plausible in an aging, polypharmacy, post-pandemic population—is proposed as the mechanistic substrate of the MVT hypothesis. We stress that these stressors differ substantially in how directly they have been linked to FQMT specifically, and Table 2 makes that evidence hierarchy explicit.

**TABLE 2:** Major co-prescribed drug classes and comorbid stressors proposed to contribute to pre-existing mitochondrial substrate depletion and tissue vulnerability, with an explicit evidence hierarchy. Evidence levels: Direct FQMT evidence (studied in FQ toxicity); Clinical FQ risk modifier (epidemiologically associated with FQ tendon injury); Indirect mitochondrial evidence (mitochondrial effect shown, not in FQ context); General biological plausibility; Speculative extrapolation.

Stressor	Mitochondrial/connective-tissue mechanism	Substrate depleted	Evidence level for FQMT	References
Corticosteroids	Independent tendon risk factor; FQ + corticosteroid combination amplifies tendinopathy hazard	Direct collagen-metabolism effect	Clinical FQ risk modifier	Chang et al. (2022)
Statins	Mevalonate pathway → CoQ10 biosynthesis block; TNF–MAPK muscle sensitization	CoQ10 (ubiquinol)	Clinical FQ risk modifier (co-medication) + indirect mitochondrial	Ganamurali and Sabarathinam, 2025; Chang et al., 2022
Renal insufficiency	Reduced FQ clearance → accumulation; established independent tendon risk	(pharmacokinetic)	Clinical FQ risk modifier	Koumou et al., 2021; Chang et al., 2022
PPIs	Luminal pH ↑ → Mg^2+^ insolubility; TRPM6/7 downregulation; impaired B12 liberation	Mg^2+^, B12	Indirect mitochondrial/biological plausibility	Gommers et al. (2022)
Metformin	CUBN-mediated ileal B12 malabsorption; rs1801222 genotype elevates risk	B12, folate	Indirect mitochondrial/biological plausibility	Baldwin et al. (2026)
NSAIDs	MPTP opening; oxidative-phosphorylation uncoupling; pharmacodynamic potentiation of FQ GABA-A antagonism	CoQ10, glutathione	Biological plausibility	Gröber (2012)
Post-COVID-19 injury	Mitochondrial fragmentation; impaired oxidative phosphorylation; skeletal-muscle mtDNA damage	(functional)	Indirect mitochondrial evidence	Molnar et al., 2024; Scheibenbogen and Wirth, 2025
Environmental toxin burden	Complex I inhibition (rotenone-class); mtDNA methylation changes at MT-COX genes	(functional/epigenetic)	Speculative extrapolation	Li et al. (2026)

TRPM6/7: transient receptor potential melastatin channels; CUBN: cubilin gene; MPTP: mitochondrial permeability transition pore; MMP: matrix metalloproteinase.

### Statins—CoQ10 depletion and TNF–MAPK sensitization

Statins inhibit HMG-CoA reductase, simultaneously suppressing cholesterol and CoQ10 biosynthesis (Littarru and Langsjoen, 2007). A pharmacological model in healthy subjects showed simvastatin 40 mg/day produced measurable mitochondrial dysfunction (37% prolongation of phosphocreatine-recovery time), partially reversed by ubiquinol (van Diemen et al., 2017). Network-pharmacology analysis identifies TNF as a principal hub through which statin-associated CoQ10 deficiency may amplify muscle injury via MAPK-mediated apoptotic signaling (Ganamurali and Sabarathinam, 2025). The clinical trial evidence for CoQ10 supplementation in statin-associated muscle symptoms is mixed and is discussed critically (Kovacic et al., 2025; Mantle et al., 2022; Kumble and Frishman, 2026). Mechanistically, the statin-exposed tissue is plausibly both CoQ10-depleted and primed for amplified inflammatory responses to a further mitochondrial insult such as an FQ.

### Proton pump inhibitors—the magnesium-depletion vector

PPI-associated hypomagnesemia is attributed to increased luminal pH reducing intestinal Mg^2+^ solubility, TRPM6/7 transporter downregulation, and microbiome disruption (Gommers et al., 2022); long-term PPI use also impairs cobalamin liberation from dietary protein (Sheen and Triadafilopoulos, 2011). Because FQs chelate Mg^2+^ within tendon tissue partly independent of serum levels, a PPI-treated patient may present with reduced tissue Mg^2+^ reserves. We note explicitly that serum magnesium is an imperfect surrogate for tissue magnesium, so this vector is mechanistically plausible but not directly demonstrated in FQMT.

### Metformin—B12 depletion and mitochondrial substrate failure

A 2026 UK Biobank genome-wide association study (n = 7,173) identified a CUBN variant (rs1801222) conferring 12.84% B12-deficiency incidence in high-risk homozygotes at 11 years of metformin use versus 6.02% in wild-type carriers—demonstrating genetically stratifiable risk (Baldwin et al., 2026). B12 deficiency can impair one-carbon metabolism and mitochondrial function (Owen et al., 2021; Green et al., 2017), potentially compounding substrate availability for repair.

### Sedentary lifestyle—PGC-1α suppression and biogenesis failure

Sedentary elderly individuals exhibit approximately 50% reductions in PGC-1α and SIRT3 versus active young controls, ∼41% lower mitochondrial respiration, and ∼30% lower cytochrome-c-oxidase activity (Joseph et al., 2012). Frailty—overlapping substantially with chronic statin, PPI, and metformin use—is associated with reduced mtDNA content and respiratory capacity and elevated ROS (Marzetti et al., 2025).

### Metabolic syndrome and obesity—lipotoxicity and mitochondrial uncoupling

Intramuscular lipid accumulation in metabolic syndrome disrupts fatty-acid β-oxidation, enhances ROS production, and drives mitochondrial dysfunction through TNF-α, IL-6, and dysregulated mitochondrial dynamics (Barazzoni et al., 2018).

### Post-COVID-19 infection—mitochondrial injury at population scale

SARS-CoV-2 can cause mitochondrial damage through interference with mitochondrial antiviral signaling, fragmentation, cytokine-mediated oxidant load, and impaired quality control (Molnar et al., 2024). In long COVID—affecting a large global population (Al-Aly et al., 2024) — mitochondrial dysfunction is described as a central driver of fatigue and exercise intolerance, with structural mitochondrial damage reported in skeletal muscle of post-COVID ME/CFS patients (Scheibenbogen and Wirth, 2025).

### Antibiotic-induced gut dysbiosis—loss of mitochondrial signaling substrates

Antibiotic treatment can shift the gut microbiome toward dysbiotic states with reduced short-chain-fatty-acid production (Lange et al., 2016). Butyrate and propionate regulate PGC-1α expression and modulate NLRP3 inflammasome and NF-κB signaling along a gut–mitochondria axis (Chauhan et al., 2025).

### Environmental toxin burden—complex I inhibition and mtDNA epigenetic effects

Rotenone-class pesticides inhibit mitochondrial complex I through a pathway conserved from *C. elegans* to human cells (Vrijsen et al., 2020). Ubiquitous endocrine-disrupting chemicals—bisphenols, phthalates, parabens—have been associated with methylation changes at mitochondrial coding genes (MT-COX1, MT-COX3) in human platelet mitochondria (Li et al., 2026; Stoccoro and Coppedè, 2021). We flag this category as the most indirect of the eight (Table 2).

## A candidate epigenetic mechanism for repeat-exposure escalation (unvalidated)

The following section presents a speculative, explicitly unvalidated hypothesis extrapolated from mtDNA-methylation evidence in non-fluoroquinolone contexts. No longitudinal D-loop methylation data exist in FQ-exposed patients. It is offered only to motivate a specific future study design, not as established biology.

Mitochondrial DNA displacement-loop (D-loop) methylation is responsive to environmental and metabolic stressors, with altered patterns reported in several disease states (Stoccoro and Coppedè, 2021; Li et al., 2026). We hypothesize—as one candidate mechanism to be tested, not as an explanation of any clinical observation—that FQ-induced oxidative stress could generate D-loop methylation changes that persist after drug discontinuation and might alter the mitochondrial transcriptional response to a subsequent exposure (Figure 2). We explicitly do not claim that D-loop methylation accounts for clinically reported repeat-exposure worsening; that clinical pattern has multiple possible explanations (cumulative tissue injury, changing host reserve, ascertainment) and remains unproven.

**FIGURE 2:**
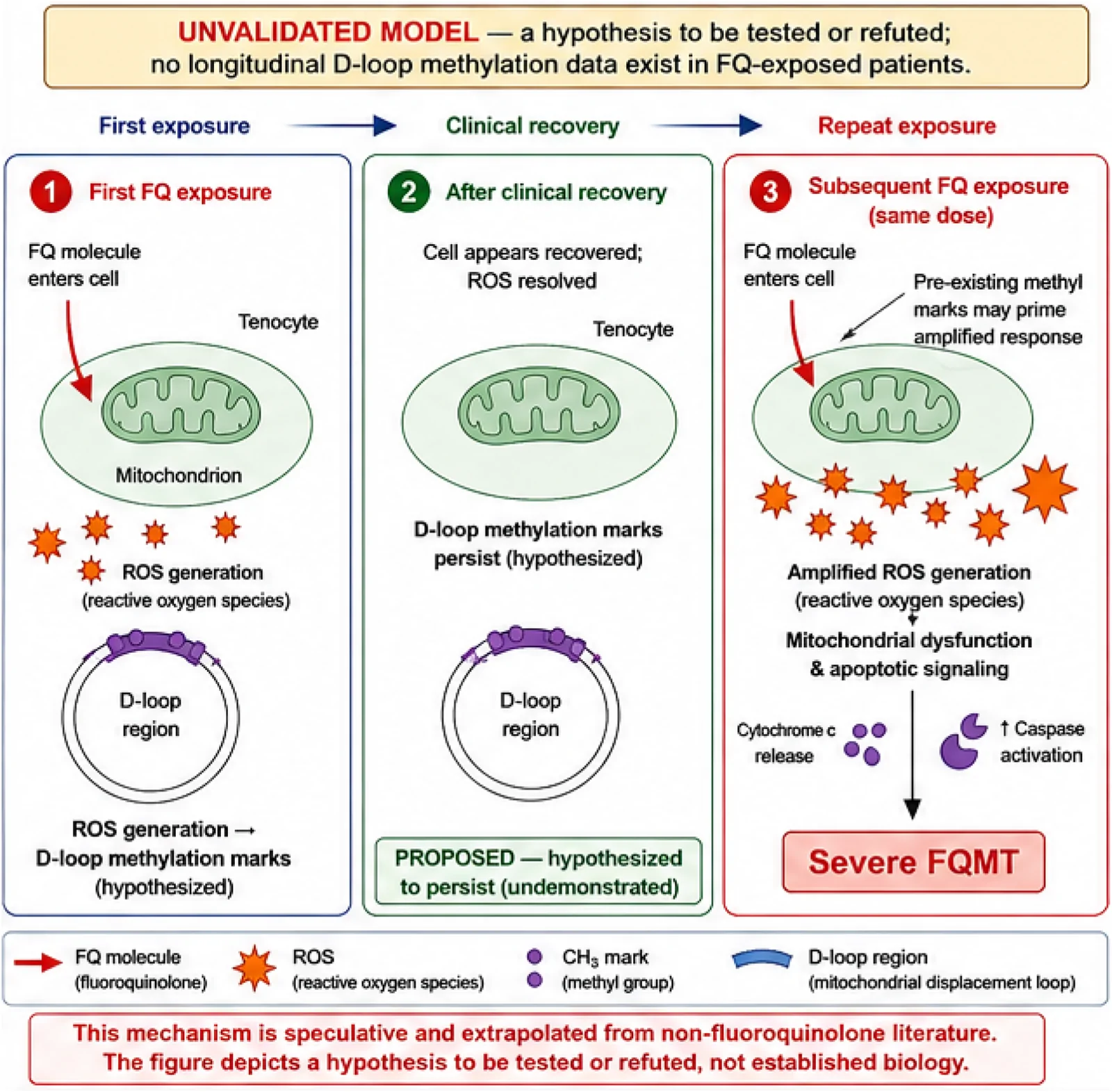
Unvalidated model—a proposed epigenetic priming mechanism for repeat-exposure escalation. Panel 1: a first FQ exposure generates reactive oxygen species and is hypothesized to produce D-loop methylation marks in tenocyte mtDNA. Panel 2: these marks are hypothesized—not demonstrated—to persist after clinical recovery. Panel 3: on a subsequent exposure, pre-existing marks are hypothesized to amplify the mitochondrial transcriptional response. This mechanism is speculative and extrapolated from non-fluoroquinolone literature; no longitudinal D-loop methylation data exist in FQ-exposed patients, and the figure depicts a hypothesis to be tested or refuted, not established biology. D-loop: mitochondrial displacement loop; ROS: reactive oxygen species; FQMT: fluoroquinolone-associated multisystem toxicity.

A frequently cited drug precedent is long-term nucleoside reverse-transcriptase-inhibitor (NRTI) exposure, associated with mtDNA D-loop, ND1, and ND4 variation and with accumulated mitochondrial dysfunction (Liang et al., 2017; Torres and Lewis, 2014). We include this precedent only to establish that drug-associated, exposure-dependent mtDNA changes are biologically possible, and we caution that the analogy is mechanistically limited: NRTI toxicity is classically driven by inhibition of mitochondrial DNA synthesis (polymerase-γ–related mechanisms), whereas the FQ mechanisms discussed here center on topoisomerase-II effects, Mg^2+^ chelation, oxidative stress, matrix injury, and tenocyte apoptosis. The two should not be equated. Testing this hypothesis would require longitudinal D-loop methylation profiling in patients before and after FQ courses, stratified by prior exposure and clinical outcome.

## The prescribing-inertia problem

The 2016 expanded FDA boxed warning was not followed by a statistically significant reduction in FQ prescription rates for the three indications it addressed (Bratsman et al., 2020). Inappropriate prescribing for uncomplicated cystitis—for which nitrofurantoin, fosfomycin, and trimethoprim-sulfamethoxazole are effective alternatives—has been reported at 89.5% across five family-practice clinics (Robinson et al., 2019), and a 2025 multimodal VA stewardship intervention achieved only partial reductions despite intensive effort (Kinyua et al., 2025).

The expanding FAERS literature—panic-attack reporting several-fold higher than comparators, psychiatric events across age groups, and ocular signals (Chau and Au, 2026; Raguram et al., 2026) — suggests the reported harm profile is broader than commonly appreciated. As emphasized, these disproportionality signals can generate hypotheses but cannot establish incidence, magnitude, temporality, or causality, and their growth over time should not be interpreted as evidence that FQMT has become biologically more severe; increased clinical awareness and reporting are sufficient to produce such growth. With that caveat, the persistent gap between class-level warnings and prescribing behavior motivates the development—and prospective validation—of individualized, patient-specific risk-assessment research tools.

## The mitochondrial vulnerability score (MVS): a candidate research instrument

We propose the MVS as a candidate research instrument for prospective evaluation—not as a clinical prescribing tool, guideline, or decision aid. Its point values are provisional and mechanistically motivated; they have not been empirically derived from, or calibrated against, FQMT outcome data, and the score has not been validated. We present it so that it can be tested, refined, or refuted (Table 3, and Prediction 1 in Table 4). Accordingly, the instrument itself contains no prescribing thresholds and no management recommendations; provisional risk strata for prospective testing are presented separately as parameters to be validated, not as decision rules.

**TABLE 3:** The mitochondrial vulnerability score (MVS): a proposed, un-calibrated candidate research instrument for prospective testing. Point values are provisional and mechanistically indicative, not empirically derived, and are accompanied by an explicit evidence level for each item. This is not a clinical guideline or prescribing tool. [speculative] = mechanism proposed but not demonstrated.

Candidate item	Provisional points	Mechanistic basis	Evidence level	Key reference(s)
Chronic renal insufficiency (eGFR <60)	+2	Reduced FQ clearance; established independent tendon risk	Clinical FQ risk modifier	Koumou et al., 2021; Chang et al., 2022
Corticosteroid use (systemic >2 weeks)	+1	Independent tendon risk; FQ + steroid amplifies hazard	Clinical FQ risk modifier	Chang et al. (2022)
Statin use (any)	+2	CoQ10 depletion; TNF–MAPK sensitization	Clinical risk modifier + indirect mitochondrial	Kovacic et al., 2025; Ganamurali and Sabarathinam, 2025
Documented Mg^2+^ deficiency (serum <0.75 mmol/L)	+2	Chelation-substrate depletion (serum imperfect surrogate)	Indirect/biological plausibility	Rosanoff et al. (2016)
Age ≥65 years	+1	Baseline decline in respiratory capacity	General biological plausibility	Marzetti et al., 2025; Niccoli and Partridge, 2012
PPI use (>3 months)	+1	Intestinal Mg^2+^/B12 malabsorption	Indirect/biological plausibility	Gommers et al. (2022)
Metformin use (any)	+1	CUBN-pathway B12 malabsorption	Indirect/biological plausibility	Baldwin et al. (2026)
Documented B12 deficiency (<200 pg/mL)	+1	Impaired one-carbon/repair substrate	Indirect/biological plausibility	Green et al. (2017)
Obesity/metabolic syndrome	+1	Lipotoxic mitochondrial uncoupling	Indirect/biological plausibility	Barazzoni et al. (2018)
Sedentary lifestyle (<150 min/wk)	+1	PGC-1α suppression; biogenesis failure	Indirect/biological plausibility	Joseph et al. (2012)
Prior COVID-19 infection	+2	Mitochondrial fragmentation; muscle mtDNA damage	Indirect mitochondrial evidence	Molnar et al. (2024)
Prior FQ course within 5 years	+2	Proposed residual D-loop epigenetic priming	Speculative extrapolation [speculative]	Stoccoro and Coppedè (2021)

PGC-1α: peroxisome proliferator-activated receptor gamma coactivator 1-alpha; CUBN: cubilin.

**TABLE 4:** Falsifiable predictions of the MVT model with proposed study designs and primary outcome measures.

Falsifiable prediction	Proposed study design	Primary outcome measure
Patients with higher MVS will have higher FQMT rates than those with lower MVS at equivalent FQ doses	Prospective observational cohort; MVS computed pre-prescription; 90-day follow-up; adjusted Cox regression	HR for FQMT per MVS-point increment
FAERS FQ musculoskeletal reports will be enriched for co-reported statin, PPI, and metformin use vs. active comparators	Disproportionality analysis (FAERS 2010–2025); active-comparator design; adjusted ROR by co-medication count	Adjusted ROR by co-medication burden (signal only; not incidence)
D-loop mtDNA methylation will differ in FQ-exposed patients vs. controls and correlate with FQMT severity	Longitudinal study; D-loop bisulfite pyrosequencing before and 6 weeks after FQ; correlation with outcomes	Δ D-loop methylation; correlation with 30-day VAS pain
Prophylactic Mg^2+^ + CoQ10 will reduce FQMT incidence in higher-MVS patients requiring FQ	Double-blind RCT; higher-MVS patients; supplement vs. placebo started before FQ; 90-day follow-up	Time to FQMT diagnosis; 30-day severity score
FQ-exposed post-COVID patients will show higher FQMT incidence than FQ-exposed non-COVID controls	Retrospective EHR cohort; propensity-matched by prior COVID-19 diagnosis	HR for FQMT, post-COVID vs. non-COVID

FAERS: FDA adverse event reporting system; HR: hazard ratio; ROR: reporting odds ratio; VAS: visual analogue scale; RCT: randomized controlled trial; EHR: electronic health record; MVS: Mitochondrial Vulnerability Score.

### Parameters to be tested in prospective validation

The following risk strata are not recommendations for practice. They are provisional parameters that a validation study would test, refine, or reject; they are listed here, separated from the instrument, precisely so that they are not read as a prescribing algorithm. In any validation study, the cut-points below should be treated as hypotheses to be optimized against observed outcomes (e.g., by ROC analysis), not as fixed decision boundaries:

- Provisional stratum A (score 0–2): hypothesized standard vulnerability—to be tested as the reference group.
- Provisional stratum B (score 3–5): hypothesized elevated vulnerability—a candidate group in which prophylactic co-interventions might be evaluated.
- Provisional stratum C (score 6–8): hypothesized high vulnerability—a candidate group for enhanced monitoring endpoints in a trial.
- Provisional stratum D (score ≥9): hypothesized very high vulnerability—a candidate group for evaluating whether alternative-antibiotic strategies reduce FQMT incidence.

### Validation cohort and biomarkers

Prospective validation would ideally use a multicenter prospective observational cohort of adults receiving a first FQ course for a defined indication (Figure 3), with pre-prescription capture of every candidate MVS item and standardized, blinded outcome adjudication using a pre-specified FQMT case definition and severity scale, over ≥90 days (and a longer arm for persistent/multisystem outcomes). Recommended baseline and follow-up biomarkers fall into three tiers: (i) accessible clinical biomarkers—serum and, where feasible, intracellular/RBC magnesium, serum B12 and methylmalonic acid, CoQ10 status, eGFR, hs-CRP; (ii) functional mitochondrial biomarkers—peripheral-blood-mononuclear-cell or platelet respirometry (spare respiratory capacity, coupled/maximal OCR), mtDNA copy number, and, where tissue is available, muscle/tendon ETC complex activities; and (iii) exploratory mechanistic biomarkers—circulating cell-free mtDNA, oxidative-stress markers (e.g., 8-OHdG, F2-isoprostanes), and, for the epigenetic priming hypothesis, D-loop methylation. A nested case–control design could apply the more resource-intensive tiers (ii–iii) to adjudicated cases and matched controls.

**FIGURE 3:**
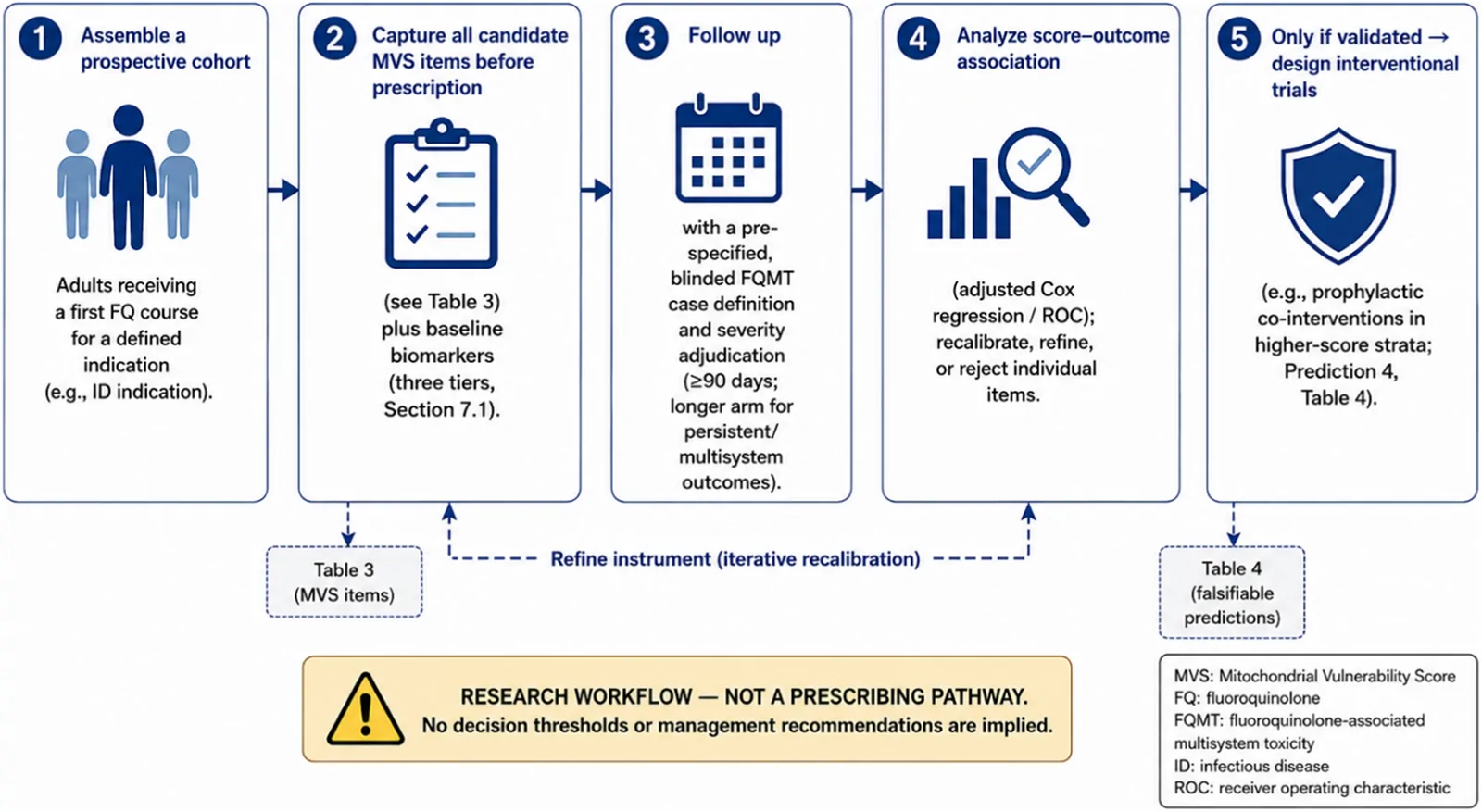
Proposed prospective-validation workflow for the mitochondrial vulnerability score (a research workflow, not a prescribing pathway). The schematic outlines how the MVS could be evaluated as a candidate research instrument: (1) assemble a prospective cohort of patients receiving a first FQ course for a defined indication; (2) capture all candidate MVS items before prescription; (3) follow patients using a pre-specified, blinded FQMT case definition and severity adjudication; (4) analyze the association between score and outcome and recalibrate, refine, or reject individual items accordingly; and (5) only if validated, design interventional trials (for example, prophylactic co-interventions in higher-score strata). No prescribing thresholds or management recommendations are implied. MVS: Mitochondrial Vulnerability Score; FQ: fluoroquinolone; FQMT: fluoroquinolone-associated multisystem toxicity; ID: infectious disease.

## Falsifiable predictions of the MVT model

Table 4 presents five falsifiable predictions, each with a proposed study design and primary outcome. These predictions are explicitly refutable; negative results would require revision or rejection of the corresponding component of the MVT hypothesis. The highest-priority near-term study—Prediction 2, a FAERS disproportionality analysis—requires no new data collection, but its interpretation is bounded by the FAERS limitations (it would test co-medication enrichment among reports, not incidence or causation).

## Discussion

### Toward possible reclassification: severe FQMT as a candidate acquired mitochondrial disorder

This subsection is academic and does not constitute a clinical or regulatory recommendation. We raise it narrowly and with appropriate caution. Current nosology classifies FQMT as a drug adverse effect. We note only that severe, persistent, multisystem FQMT—when it occurs—shares some surface features with recognized acquired mitochondrial disorders (persistence after withdrawal, multisystem involvement, exacerbation by metabolic stress), and that this observation may merit future deliberation. We do not assert that FQMT is such a disorder.

Crucially, the field currently lacks the elements required to support any such classification, and we specify them here. Before severe FQMT could be considered an acquired mitochondrial disorder, the following would, at minimum, need to be established: (i) reproducible, quantified mitochondrial functional defects in affected tissue or accessible surrogate cells (e.g., reduced spare respiratory capacity or ETC complex activity) that distinguish cases from appropriate controls; (ii) demonstrable mtDNA changes (copy-number, deletion, sequence, or methylation) associated with the phenotype; (iii) tissue-specific pathology (biopsy or imaging) or a validated omics signature; and (iv) validated circulating biomarkers with defined sensitivity and specificity, together with consensus, FQMT-specific diagnostic criteria developed through a multidisciplinary process. We also note counterarguments: FQMT has not been characterized by polymerase-γ inhibition (the FQ mechanisms are topoisomerase-II effects and Mg^2+^ chelation, not direct mtDNA-synthesis disruption); most FQMT is self-limiting; and alternative explanations (immune-mediated neuropathy, connective-tissue effects, endocrine disturbance) cannot be excluded. We therefore frame reclassification strictly as a question for future evidence, not a proposal for present adoption.

### Restoring mitochondrial reserve: candidate research directions

The IRP paradigm shifts the therapeutic question from palliating FQMT symptoms toward restoring the mitochondrial reserve capacity hypothesized to be insufficient at the time of exposure—where ‘restoration’ means moving the measurable reserve parameters defined (spare respiratory capacity, ETC substrate and antioxidant content, cofactor status, biogenic potential) toward normal. We stress that the interventions in Table 5 are candidate approaches for preclinical and clinical testing, not recommended management strategies; FQMT-specific interventional evidence is limited or absent for all of them. Dose ranges are not given in the main text; where provided, they appear only in the Supplementary Material and are labeled as hypothesis-informed and not for clinical use.

**TABLE 5:** Candidate restorative targets and interventions for future FQMT research, organized by mechanism. All are hypothesis-generating and require FQMT-specific preclinical/clinical validation; none is a management recommendation. Doses are intentionally omitted from the main text.

Restorative target	Candidate interventions	Mechanistic rationale + evidence level (for FQMT)
ETC substrate repletion	CoQ10 (ubiquinol); MitoQ	Restores electron-carrier capacity; MitoQ superior tenocyte protection *in vitro* (Lowes et al., 2009); CoQ10 clinical data in SAMS are mixed. Hypothesis-informed for FQMT.
Mg^2+^ repletion	Magnesium (oral)	Aims to counter FQ Mg^2+^ chelation in connective tissue; mechanistic + indirect only; serum a poor tissue surrogate (Gommers et al., 2022; Rosanoff et al., 2016). Hypothesis-informed
Glutathione regeneration	N-acetylcysteine	Glutathione precursor; *in vitro* reduction of FQ-induced ROS in tenocytes (Pouzaud et al., 2004; Lowes et al., 2009). Hypothesis-informed
Collagen-matrix support	Collagen peptides + vitamin C + graded loading	Substrate/cofactor for collagen synthesis; vitamin C RCT increased P1NP with pre-exercise gelatin (Shaw et al., 2017; Noriega-González et al., 2022). Indirect (non-FQ)
One-carbon metabolism	Vitamin B12 (methylcobalamin)	Corrects metformin/PPI-associated deficiency; restores repair substrate (Green et al., 2017). Evidence-based for deficiency reversal; hypothesis-informed for FQMT.
Mitochondrial biogenesis	Graded eccentric loading (rehabilitation)	Activates PGC-1α; Level-1 evidence for tendinopathy rehabilitation generally (non-FQ). Not to be applied during acute injury without assessment

ETC: electron transport chain; SAMS: statin-associated muscle symptoms; P1NP: procollagen I N-terminal propeptide.

#### Mitochondria-targeted antioxidants: the MitoQ evidence

Among antioxidant strategies, mitochondria-targeted compounds merit specific attention within the IRP framework. MitoQ—a triphenylphosphonium-conjugated CoQ10 analogue that accumulates in the mitochondrial matrix—showed superior protection over a non-targeted antioxidant in preserving tenocyte mitochondrial membrane potential and reducing oxidative stress in FQ-exposed human Achilles tenocytes in direct *in vitro* comparison (Lowes et al., 2009). This remains, to our knowledge, the only published tissue-level evidence addressing mitochondria-targeted therapy in an FQMT model, and it is *in vitro*; it illustrates, but does not establish, the IRP principle that restoration may require delivery to the injured subcellular compartment. It is not evidence of clinical benefit.

#### Prophylaxis versus treatment: when might reserve support be given?

Because the MVT hypothesis locates risk in reserve that is already low before the drug is given, it raises a logically prior question to treatment of established injury: could reserve-supporting measures be more effective if given prophylactically—during or immediately before an FQ course in higher-vulnerability patients—rather than only after toxicity appears? Mechanistically this is plausible for the cofactor-repletion measures whose depletion is the proposed vulnerability (for example, correcting a documented magnesium or B12 deficiency before exposure, or co-administering CoQ10 in a statin-treated patient), because these aim to raise the threshold before the insult rather than to repair damage after it. This is precisely the intent of Prediction 4 (Table 4), a double-blind RCT of prophylactic Mg^2+^ + CoQ10 in higher-MVS patients requiring an FQ. We emphasize the necessary cautions: no trial has yet tested prophylactic supplementation against FQMT; supplements are not without risk or interactions; and the appropriate and evidence-based action for a documented deficiency (e.g., hypomagnesemia) is its correction on its own merits, independent of this hypothesis. Prophylaxis against FQMT specifically should be regarded as a research question, not current practice.

#### Long-term speculative direction: cell-based regenerative therapy

As a long-term and frankly speculative direction only, cell-based therapies with intrinsic regenerative and mitochondrial-transfer properties—for example, Multilineage-differentiating Stress Enduring (MUSE) cells, which can donate functional mitochondria to stressed cells via tunneling nanotubes and exert paracrine anti-apoptotic and matrix-protective effects—have a mechanistic profile that overlaps conceptually with FQMT pathophysiology. We deliberately do not detail delivery routes, dosing, or administration strategies, because there are no clinical data for such therapies in FQMT and any such detail would be premature. We mention this direction solely to flag it for future preclinical investigation; it is not a proposed treatment.

### Counterbalancing evidence and alternative explanations

Consistent with a balanced synthesis, we set out the principal evidentiary tensions in this framework. First, the clinical evidence for CoQ10 is mixed: while a 2025 systematic review/meta-analysis reported benefit for statin-associated muscle symptoms (Kovacic et al., 2025), other syntheses have been more equivocal and heterogeneity is high (Mantle et al., 2022; Kumble and Frishman, 2026), and there is no FQMT-specific clinical trial; CoQ10’s role here is therefore mechanistic and extrapolated. Second, magnesium status is difficult to measure: serum magnesium correlates poorly with tissue and intracellular stores, so “normal” serum values do not exclude tissue depletion and, conversely, the clinical significance of the proposed tissue-chelation vector cannot be confirmed by serum testing alone (Rosanoff et al., 2016). Third, the clinical relevance of mtDNA (including D-loop) methylation remains uncertain: associations exist across disease states (Stoccoro and Coppedè, 2021; Li et al., 2026), but causal and FQ-specific data are absent, and this hypothesis is explicitly labeled speculative. Fourth, not all FQ toxicity is mitochondrial: important mechanisms are non-mitochondrial or only partly so—notably GABA-A antagonism/NMDA activation underlying CNS effects (Radkowski et al., 2025), direct MMP-mediated collagen proteolysis, and possible immune-mediated contributions—and a purely mitochondrial reading would be incomplete. The MVT hypothesis is intended to complement, not supplant, these mechanisms.

### Limitations

We acknowledge the following limitations explicitly.

- The MVT hypothesis is a mechanistic synthesis. Direct evidence for convergent, synergistic mitochondrial impairment across all eight stressor categories simultaneously in the same patient is lacking; comprehensive mitochondrial phenotyping in polypharmacy patients is needed.
- We do not demonstrate that FQMT incidence or severity has increased over time. This is presented throughout as a testable hypothesis, not a finding; no formal epidemiological trend analysis with standardized case definitions and prescription denominators is presented or, to our knowledge, exists.
- The MVS is an un-calibrated candidate research instrument. Its point values and the provisional strata are not empirically derived and are not for clinical use.
- The epigenetic priming hypothesis is speculative and extrapolated from non-FQ literature; no longitudinal D-loop methylation data from FQ-exposed patients exist, and the NRTI analogy is mechanistically limited.
- The reclassification discussion is preliminary academic deliberation, conditioned on evidence that does not yet exist; no regulatory or clinical recommendation is implied.
- All restorative and prophylactic interventions (Table 5; Supplementary Table S1) are hypothesis-informed candidates for testing, not clinical guidance. Dosing information, provided only in the Supplementary Material, must not substitute for individualized clinical assessment.
- This is a narrative synthesis, not a systematic review; it did not apply formal inclusion criteria or risk-of-bias assessment, and selection bias in the cited literature cannot be excluded.

## Conclusion

The clinical observation that some patients develop severe or persistent fluoroquinolone-associated toxicity while many tolerate the same drugs is not fully explained by the drug alone. We hypothesize that part of this variability reflects differences in host mitochondrial reserve, and that converging iatrogenic, nutritional, infectious, and environmental stressors may lower that reserve in a substantial subset of contemporary patients—a proposition we frame as testable rather than demonstrated. The MVS is offered as a candidate research instrument; the epigenetic-priming mechanism is speculative but falsifiable; the acquired-mitochondrial-disorder discussion is academic and conditioned on evidence not yet available.

Table 4 provides a structured research agenda of five falsifiable predictions. The highest-priority near-term study is a FAERS disproportionality analysis correlating FQ musculoskeletal reports with co-reported statin, PPI, and metformin use—interpretable, within FAERS limitations, as a signal-generating test of co-medication enrichment.

Viewed through the lens of integrative and regenerative pharmacology, the practical implication is twofold: first, that pre-prescription risk assessment tools deserve prospective development and testing; and second, that restoring the biological substrate hypothesized to fail—mitochondrial reserve—is a research direction worth pursuing alongside prescribing reform. The core message is a question for future evidence to answer: before prescribing a fluoroquinolone, might it matter not only whether the drug is indicated, but whether this patient has the mitochondrial reserve to tolerate it—and if not, whether that reserve can be supported?

## Data Availability

The original contributions presented in the study are included in the article/Supplementary Material, further inquiries can be directed to the corresponding author.
