# Supplementary material for: Depleted before the dose: the mitochondrial vulnerability threshold hypothesis for fluoroquinolone-associated multisystem toxicity
Source: Front Pharmacol. 2026 Sep 23;17:1905942. doi: 10.3389/fphar.2026.1905942 (PMC13643692; doi:10.3389/fphar.2026.1905942)
Supplement: Supplementary file 1 [file Table1.pdf]

Supplementary Table S1. Hypothesis-informed restorative interventions for the mitochondrial-reserve framework, with proposed (non-validated) dose ranges and evidence grading. IMPORTANT: All dose ranges below are hypothesis-informed, are extrapolated from non-FQMT contexts, and are NOT clinical recommendations, prescribing guidance, or treatment protocols; no FQMT-specific dosing has been validated in any prospective trial. Interventions are candidates for preclinical/clinical testing only (see Table 5). ETC: electron transport chain; SAMS: statin-associated muscle symptoms; P1NP: procollagen I N-terminal propeptide; RBC: red blood cell; FQMT: fluoroquinolone-associated multisystem toxicity.

**Intervention**	**Restorative target / mechanism**	**Proposed dose range (hypothesis-informed — NOT clinical advice)**	**Evidence level (FQMT / general)**	**Key reference(s)**	**Safety / practical notes**
CoQ10 (ubiquinol)	ETC substrate repletion; restores complex I/II electron transfer; antioxidant	~100–300 mg/day (ubiquinol); SAMS studies commonly ~200 mg/day	Indirect / mixed clinical (SAMS); hypothesis-informed for FQMT	Kovacic et al., 2025; Mantle et al., 2022; Kumble & Frishman, 2026; Littarru & Langsjoen, 2007; Lowes et al., 2009	Generally well tolerated; may reduce warfarin effect; take with fat-containing meal. Clinical benefit in SAMS is mixed.
MitoQ (mitochondria-targeted)	Matrix-targeted antioxidant; preserves membrane potential; reduces mitochondrial ROS	~10–20 mg/day (typical supplement range)	Direct in vitro (FQ tenocytes); no clinical FQMT data	Lowes et al., 2009	Only tissue-level FQ evidence is in vitro; no clinical efficacy or long-term safety data in FQMT.
Magnesium (glycinate or malate)	Repletes divalent-cation pool depleted by FQ chelation; collagen/neuromuscular support	~200–400 mg elemental/day (divided)	Mechanistic + indirect; hypothesis-informed for FQMT	Gommers et al., 2022; Rosanoff et al., 2016	Diarrhea at higher doses; caution in renal impairment. Serum Mg is a poor surrogate for tissue Mg.
N-acetylcysteine (NAC)	Glutathione precursor; restores antioxidant buffering; reduces FQ-induced ROS in vitro	~600–1200 mg/day	In vitro (FQ tenocytes); hypothesis-informed for FQMT	Pouzaud et al., 2004; Lowes et al., 2009	Generally well tolerated; GI upset possible; theoretical interaction with nitrates.
Vitamin B12 (methylcobalamin)	Restores one-carbon metabolism / methylation and repair substrate; corrects PPI/metformin-related deficiency	~1000 µg/day oral (or per established deficiency-correction protocol)	Evidence-based for deficiency reversal; hypothesis-informed for FQMT	Green et al., 2017; Owen et al., 2021; Baldwin et al., 2026	Correct documented deficiency on its own merits; parenteral route if malabsorption. Low toxicity.
Collagen peptides + Vitamin C	Collagen-matrix substrate/cofactor; vitamin C is cofactor for prolyl/lysyl hydroxylase	~15 g hydrolyzed collagen/gelatin + ~50 mg (up to ~500 mg) vitamin C, ~30–60 min before loading	Indirect clinical (non-FQ; increased P1NP); hypothesis-informed for FQMT	Shaw et al., 2017; Noriega-González et al., 2022	Timing before graded loading may matter; food product, low risk. No FQMT-specific trial.
Graded eccentric loading (rehabilitation)	Mechanotransduction + PGC-1α–driven biogenesis; tendon remodeling	Progressive eccentric protocol (individualized); begin low-load; avoid high-impact during acute phase	Level-1 evidence for tendinopathy generally (non-FQ); hypothesis-informed for FQMT	(tendinopathy rehabilitation literature)	Not to be applied to an acutely inflamed/at-risk tendon without assessment; supervise progression.
